# Towards a more practical attention bias test to assess affective state in sheep

**DOI:** 10.1371/journal.pone.0190404

**Published:** 2018-01-02

**Authors:** Jessica E. Monk, Rebecca E. Doyle, Ian G. Colditz, Sue Belson, Greg M. Cronin, Caroline Lee

**Affiliations:** 1 Agriculture and Food, CSIRO, Armidale, NSW, Australia; 2 School of Environmental and Rural Science, University of New England, Armidale, NSW, Australia; 3 Animal Welfare Science Centre, University of Melbourne, Parkville, VIC, Australia; 4 Faculty of Science, University of Sydney, Camden, NSW, Australia; Leiden University, NETHERLANDS

## Abstract

Tests for attention bias potentially offer more rapid assessment of affective state in animals than existing cognitive methods. An attention bias test has previously been developed for sheep and validated as a measure of anxious states. The 3 minute test assessed behavioural responses of sheep in an enclosed arena after brief exposure to the threat of a dog. Experiment 1 of the current study aimed to refine the previously developed method, removing the need for a habituation period and shortening the test duration. Sheep were given either an anxiolytic drug, an anxiogenic drug or a control treatment prior to testing to induce contrasting affective states. Differences in behaviour were found between the treatment groups within the first 45s of the test, indicating the original test duration could be shortened from 180 s. During testing, 36 of 40 animals in the control and anxiolytic groups ate the novel feed offered in the test, indicating it is not necessary to habituate animals to a feed container. Experiment 2 aimed to confirm the responses measured in the test were primarily towards the dog rather than other aspects of the test environment. Sheep exposed to an empty window at the beginning of the test behaved differently to those which were exposed to a dog, indicating sheep behaviour in the test is at least partially a response to the dog. A third group of sheep were also tested with the dog immediately after having small data loggers attached to their necks. Behaviour of these sheep did not differ from the sheep tested without loggers, indicating data logger attachment did not impact their behaviour in the test. In both experiments, treatments did not appear to modify activity (zones crossed), which we propose indicates the test was primarily detecting valence of the affective state rather than arousal.

## Introduction

As animal welfare becomes an increasingly important consideration for society, we need to develop more practical measures of welfare which take into account the emotional or affective states of animals. Affective state is currently understood as a position of the animal within an “affective space” delineated by axes described as valence and arousal [1,2]. Arousal describes the physiological activation of the state while valence describes whether the state is hedonically positive or negative. Negatively valenced states may be particularly strong indicators of poor welfare, however it can often be difficult to determine the valence of an affective state using behavioural and physiological indicators. For example heart rate can be used as an indicator of arousal but increases in heart rate occur across a range of emotional valences which may be positive (e.g. meeting a sexual partner), negative (e.g. exposure to a predator) or neutral (e.g. increased locomotion) [3]. An alternative approach for assessing valence is to use a concept termed cognitive bias, where the affective state of an animal alters the way it processes information, which in turn affects the behavioural responses of the animal to its environment [3]. By measuring the variation in responses between individuals or treatment groups in defined behavioural test paradigms we can make inferences about the underlying affective state of an animal [4]. The form of cognitive bias most widely studied in non-human animals thus far is judgement bias, in which the emotional state of the animal influences its interpretation of ambiguous situations [3]. Judgement bias tests have been used to study both positively and negatively valanced affective states in a range of animal species including rats, dogs and sheep [3,5,6]. The majority of judgement bias tests follow a paradigm developed by Harding et al. [7], which initially involves training animals to discriminate between and respond to a positive and a negative stimulus. The requirement for a training period means typical judgement bias tests are impractical as applied measures of welfare for livestock in production environments. Furthermore, not all animals successfully learn the test procedure, and thus a portion of the population of interest need to be excluded from assessment of judgement bias. A further limitation of the judgement bias methodology has been the difficulty of differentiating between the influence of arousal from the influence of valence on the response of the animal to ambiguous cues.

Another type of cognitive bias called attention bias is the tendency to process certain types of information before others [8], and may offer a more rapid method for the assessment of certain affective states in animals [3]. In human studies, individuals in high states of anxiety show greater attentional biases towards threatening stimuli than non-anxious individuals [9,10]. Attention biases have also been found in non-human primates [11], starlings [12] and sheep [13]. Starlings denied access to water baths responded to an alarm call with increased vigilance and less willingness to feed than those which were able to bath. These findings were interpreted as birds being more anxious due to compromised flight ability and consequently directing more attention towards the threatening cue. This test was adapted for use with sheep, where the threatening cue was the presence of a dog for 10 s at the beginning of the test [13]. The test was validated as a measure of anxiety by pharmacologically manipulating the anxious states of sheep. Sheep in an induced anxious state responded with increased vigilance, less willingness to feed and paid more attention to the previous location of the dog than sheep in a reduced anxious state. While the method was more rapid than existing judgement bias tests, it still required prior training to familiarise sheep to a feed bucket and the duration of the test was 3 min per animal which may limit its use in applied contexts. Removal of the need to train sheep to the bucket and reduction in the duration of the test would provide the basis for a more practical method, potentially applicable in a range of contexts such as on-farm. Additionally, Lee et al. [13] did not include a control group that were not exposed to the dog, and thus it was unclear whether the responses measured in the attention bias test were toward the dog or due to another aspect of the test such as isolation or novelty. It was suggested further use of the attention bias test should include a treatment without the dog to address this question.

The current study aimed to refine and further validate the attention bias test developed by Lee et al. [13], to measure affective states in sheep. Experiment 1 aimed to make the test more practical by shortening the duration and eliminating the need for training. We hypothesised that sheep would be willing to eat a novel food in a novel environment and that differences in behaviour between the treatment groups would be detectable in under 3 min. Experiment 2 aimed to confirm that the responses being measured during the test were directed towards the threat. We hypothesised that sheep exposed to an empty window would behave differently to those exposed to a dog, indicating the observed behaviours were at least partially due to the presence of a threat. A secondary aim for experiment 2 was to investigate whether attachment of small data loggers to the animals immediately prior to testing would alter their behaviour in the test. It was hypothesised that behaviour of animals in the Logger group would not differ from the control group, creating opportunities for automation of behavioural measurement without the need for habituation. Both experiments assessed general activity of sheep as a measure of arousal in addition to behavioural measures of attention bias.

## Materials and methods

### Animal ethics

The protocol and conduct of the experiments were approved by the CSIRO McMaster Laboratory Animal Ethics Committee and the University of New England Animal Ethics Committee, under the New South Wales Animal Research Act 1985.

### Experiment 1

#### Animal and treatment details

Sixty 5-month-old castrated male Merino lambs, born and raised at pasture, with average bodyweight 29.9 ± 3.3 kg were used in this experiment. Sheep had prior experience of supplementary feeding with oaten chaff and a pelleted ration containing comminuted lucerne, but had never been supplementary fed with hay. All sheep had undergone routine handling previous to the current experiment and were therefore familiar with the presence of humans. The sheep were randomly allocated to one of three treatment groups, balancing for bodyweight (*n* = 20 per treatment): 1) anxiolytic (Diazepam, 0.1 mg/kg i.v.), 2) anxiogenic (meta-Chlorophenylpiperazine (m-CPP), 2 mg/kg i.m.) and 3) Control (receiving saline i.m.). These dose rates have been used on sheep in previous studies to alter emotional states with no observable adverse impact on the animals for diazepam [14] or m-CPP [13,15]. m-CPP is a serotonin-2A (5-HT_2A_) receptor agonist [16,17] while diazepam works through activation of GABAergic receptors [14,17]. Drugs were administered 30 min prior to testing. It was expected that m-CPP and diazepam would increase and decrease anxiety respectively, causing changes in vigilance and feeding behaviours during the test, but that they would not have a sedative effect on the animals (as indicated by no differences in locomotory behaviour and vocalisations between groups).

#### Attention bias testing

**Fig 1:**
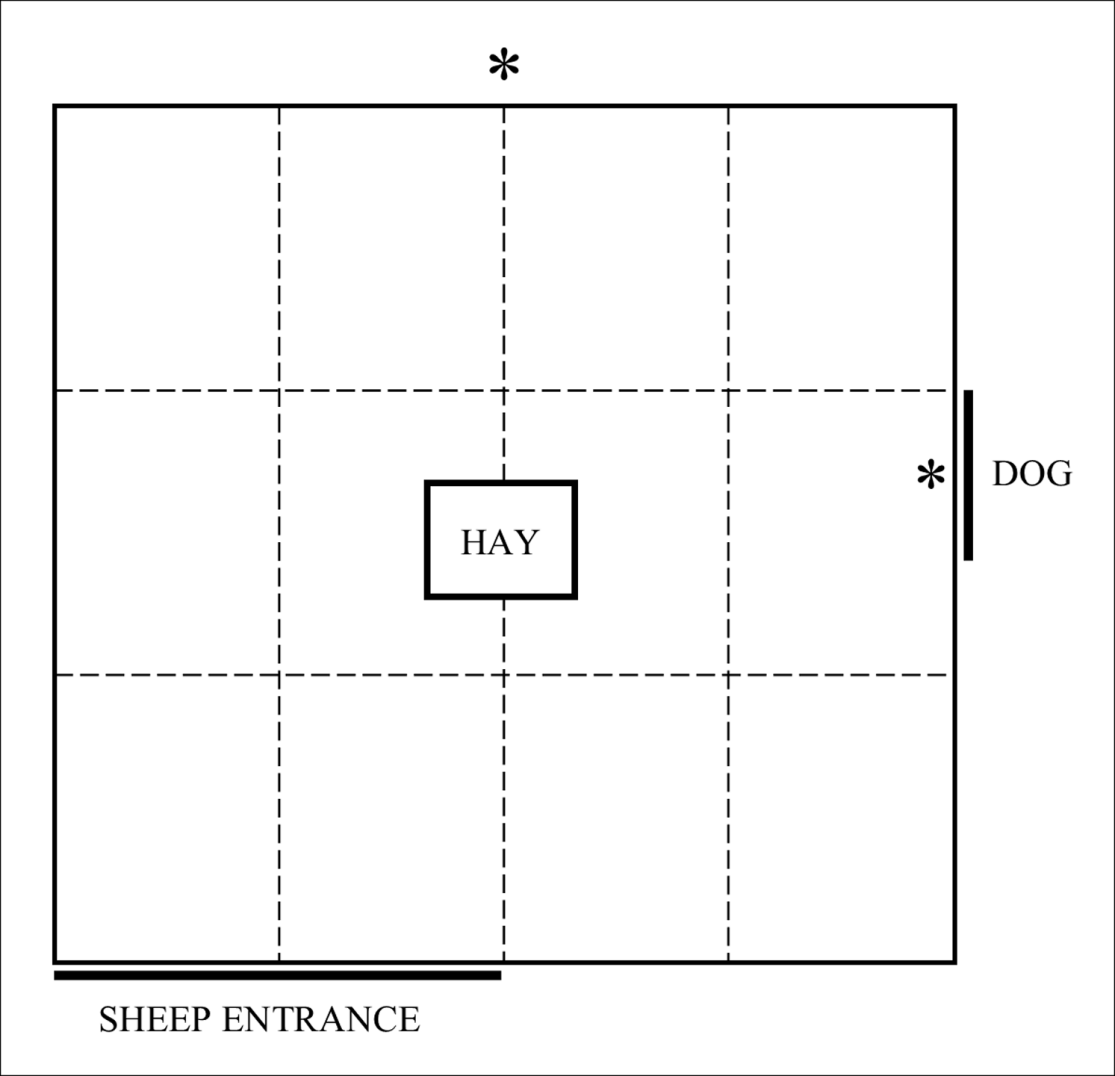
Diagram of the attention bias test arena comprising a 4 x 4.2 m yard with opaque walls 1.8 m high and hay placed in the centre. Dashed lines represent a 1 x 1.4 m grid painted on the ground. “*” denotes the positions of 2 cameras. A dog was visible for the first 3 s of the test, then the window was covered.

The current study used the same testing arena (Fig 1 and S1 Video) and treatment injection protocols as Lee et al. [13]. The test arena comprised a 4 x 4.2 m yard with opaque walls 1.8 m high. On one side of the arena, a retractable opaque cover was placed in front of a window (77 cm x 58 cm) behind which a dog (kelpie cross border collie) was located. At the beginning of the test the dog sitting quietly was visible to the sheep through the window. Once a sheep entered the arena, the door was shut behind it, and a timer was started when the sheep made visual contact with the dog. After 3 s, the opaque cover was lowered in front of the window then the dog was removed. The cover took 3 s to lower such that the dog window was fully covered with no part of the dog visible by approximately 6 s. The test ended 3 min after the sheep had first made visual contact with the dog. Animals were tested individually in a random order, ensuring equal distribution of treatments across the day. Prior to testing, feed was withheld from sheep overnight to minimise variation in appetite during testing, but sheep were given ad lib access to water.

The attention bias test used in the current study differed from that used by Lee et al. [13] in two key ways. Firstly, as the current study aimed to remove the habituation period used in the original protocol, sheep were presented with approximately 1.5 kg of lucerne hay at the centre of the test arena rather than a familiar bucket containing feed. It was expected sheep would be more likely to eat hay without training than pellets from an unfamiliar bucket, even though hay was a novel feed for this group of animals. Second, the period of exposure to the dog was shortened from 10 s in the previous study to 3 s in the current study to reduce the total test duration. It was expected 3 s would be long enough for sheep to recognise the dog as a potential threat.

#### Behavioural measurements

Latency to eat from first visual contact with the dog and vocalisations were recorded on the day of testing by observers blind to the treatment groups (one observer per behaviour). Duration of vigilance behaviour, duration of attention towards the window (attention to threat), total time spent eating and number of zones (grids) crossed were later collated from video footage by an observer blind to the treatment groups. To determine whether the test could be shortened, duration of vigilance, latency to eat, attention to threat and zones crossed were also determined for a 45 s time period after behavioural observations began. This time period was the shortest of 30, 45 and 60 s time periods to give significant results in preliminary data analysis.

Vigilance was defined as having the head at or above shoulder height [13,18]. If the sheep shook their head or entire body, this was not considered vigilance behaviour, regardless of head position relative to shoulder position. Attention towards the threat was defined as the amount of time spent with the head oriented toward the closed dog window. Attention to threat was measured for the first 60 s of the test [13]. An eating event started when the sheep began consuming the hay. The eating event continued while the sheep was chewing provided that the head stayed within approximately 20cm of the feed and the sheep remained non-vigilant. Once the sheep became vigilant or moved away from the hay, this was considered to be the end of the eating event, even if the sheep continued chewing. A sheep was considered to have crossed one zone (marked grid section, Fig 1) when both front legs were placed into a new zone or one was placed in the zone and the other was on the line. Sheep could simultaneously be vigilant, attentive to the window and crossing zones. Feeding behaviour was mutually exclusive with vigilance and zone crossing. Examples of each behaviour are given in the supporting information (S1 Video).

### Experiment 2

#### Animal and treatment details

Sixty 12-month-old castrated male Merino lambs with average bodyweight 38.5 ± 3.1 kg were used in this experiment. The lambs were born and raised at pasture with minimal exposure to humans beyond routine management practices and no experience with the attention bias test. Prior experience of supplementary feeding was unknown for this group. The sheep were randomly allocated to one of three treatment groups (*n* = 20 per treatment): 1) dog in window at commencement of test as described for experiment 1 (Dog), 2) no dog in window at the beginning of the test (No-dog) and 3) dog in window at beginning of test and sheep fitted with data loggers attached to their necks (Logger).

#### Data loggers

HOBO^®^ Pendant G acceleration data loggers were used (dimensions: 58 mm x 33 mm x 23 mm, weight: 18 g) (Onset Computer Corporation, Pocasset, MA, USA). The HOBO^®^ Waterproof Shuttle and HOBOware^®^ Pro software (version 3.7.8) were used for programming and reading the HOBO loggers (Onset Computer Corporation, Pocasset, MA, USA). Data loggers were programmed to record tilt and acceleration at a logging interval of 0.25 s (4 Hz, measurement range: ±3 gravitational force (*g*); accuracy: ±0.075 *g* at 25°C). Immediately prior to testing, loggers were activated using a magnet and then attached to the back of the neck of the sheep using a small strip of Velcro^®^. Velcro^®^ provided a fast, easy method of attachment, requiring only a short period of restraint and a small point of contact with the sheep when compared to other methods such as a collar or halter. Wool on the back of the neck was parted and the logger was nestled as close to the skin as possible so that movement of wool had minimal impact on logger angle. Sheep in all groups had been shorn 5 months prior to testing. The current paper does not discuss the suitability of these data loggers for measuring vigilance, it reports on whether attachment of the loggers altered animal behaviour during testing.

#### Attention bias testing

The Dog group underwent the attention bias test as described for experiment 1. The No-dog group underwent the same test, however sheep were exposed to an empty window instead of a dog at the beginning of the test. The opaque cover was lowered 3 s after the sheep had looked toward the empty window, unless it was unclear when the sheep looked at the window, in which case the cover was lowered after 10 s. The Logger group underwent the test as for the Dog group with exposure to the dog, however sheep had data loggers attached immediately prior to testing. The same operator attached all data loggers, keeping the location of the logger on the neck consistent between animals. Across all treatment groups, animals were caught in a predetermined order and moved to the entrance of the attention bias test prior to testing. Sheep in the Logger group were held at the entrance for up to 30 s longer than sheep in other treatment groups while the logger was attached. The current study therefore aimed to determine whether the combined effect of additional restraint time and presence of the logger during testing impacted sheep behaviour during the test.

#### Behavioural measurements

The same behaviours were measured for experiment 2 as for experiment 1, except for vocalisations which were not recorded, after vocalisations were found to be non-significant in experiment 1 (see Results section). Additionally, whether the animal sniffed the dog window was recorded. Behaviours were collated from video footage using The Observer XT 12.0 (Noldus Information Technology). Each of the behaviours were recorded by a single observer blinded to treatment group. To determine whether treatment effects were evident for a shortened version of the test, duration of vigilance and latency to eat were also determined for 60 s and 45 s time periods after behavioural observations began.

### Statistical analysis

Data were analysed in R version 3.2 [19]. P values less than 0.05 were considered to be significant, values where 0.1>P>0.05 were considered a tendency towards significance.

#### Experiment 1

Vigilance data were analysed by Kruskal-Wallis non-parametric one-way analysis of variance (ANOVA) as the parametric model residuals did not meet normality assumptions and could not be improved by transformation [20]. Normality of parametric model residuals were checked using visual assessment of normal Q-Q plots and the Shapiro-Wilk test of normality. Post hoc multiple comparison tests were performed using the package pgirmess [21]. Attention to threat data met normality assumptions and were analysed using a one-way ANOVA, fitting treatment and test order as fixed effects. Test order was not found to be a significant predictor and was subsequently dropped from the model. Zones crossed and vocalisations were analysed using generalised linear models with a quasi-poisson distribution, fitting treatment and test order as fixed effects. Test order was found to be non-significant and was dropped from both models. Zones crossed data were analysed in the same way for the 45 s time period. Use of a quasi-poisson distribution was necessary as data were found to be over-dispersed, violating the assumptions for a poisson distribution.

Latency to eat data were analysed with Cox’s proportional hazards model using survival analysis [22,23]. Any animal that failed to eat within 180 s was deemed as a censored result, recorded as a ‘survival’ incidence in the traditional way survival analysis is used. A two stage approach was needed because none of the m-CPP sheep ate the feed during the test, and so no hazard function could be predicted for this group. Firstly, the ‘survdiff’ function was used to assess differences between the survival curves for each of the three drugs. This function generates a log-rank test that compares the curves. A cox proportional hazards model was then conducted on the Diazepam and Control groups only. This method considers explanatory variables that affect the hazard of an event happening. From the fitted model, hazard ratios can be predicted to investigate the effects of different factors on whether or not an animal was likely to eat the feed. Hazard ratio values are positive values ranging from zero to infinite. A hazard ratio of >1 indicates a higher likelihood of eating the feed compared with the reference level for each categorical explanatory variable. Values between 0 and 1 indicate a lower likelihood of eating the feed compared with the reference level. Note that the use of the term hazard in survival analysis does not necessarily imply a deleterious outcome and, in this study, the hazard refers to the sheep eating the feed. The survival analyses were performed in the same way for the 45 s time period. Time spent eating was analysed by Kruskal-Wallis non-parametric one-way ANOVA.

#### Experiment 2

Data for vigilance, attention to threat and time spent eating were analysed in the same way as for experiment 1. Latency to eat data did not require a two stage approach and were analysed using only the cox proportional hazards model as described for experiment 1, fitting treatment and test order as fixed effects. Analyses were performed in the same way for the 60 and 45 s time periods for vigilance and latency to eat.

Zones crossed data were analysed using a generalised linear model with a quasi-poisson distribution as described for experiment 1, fitting treatment and test order as fixed effects. Test order was retained in the model. Two strong outliers were identified in the zones crossed data, one from the Dog group and one from the Logger group (80 and 96 zones crossed respectively, overall mean was 22). While these appeared to be valid responses from the sheep, they had high leverage within the dataset and so the zones crossed data were analysed twice, once including these outliers and once excluding them. Two-sided Fisher’s exact tests were conducted to compare how many animals in each treatment group sniffed the dog window during the test.

## Results

### Experiment 1

Differences were found between treatment groups for vigilance and attention to threat (Table 1). The m-CPP group spent more time displaying vigilant behaviour than the other groups. The Diazepam group displayed the lowest vigilance, however this was not significantly lower than the Control group (observed difference 7.2 < critical difference 13.2). The Diazepam group spent the least amount of time looking towards the dog window, while the m-CPP and Control groups did not differ. These findings were consistent for the 45 s test. There was no effect of treatment on zones crossed during the 180 s test, however animals in the Diazepam group crossed fewer zones during the first 45 s of the test (Table 1). There was no effect of treatment on vocalisations (*X*^*2*^ = 3.22, df = 2, P = 0.2).

**Table 1:** Mean ± s.e.m. behavioural responses of sheep during the attention bias test in experiment 1.

Behavioural measure	Diazepam	Control	m-CPP	Test value	P value
Vigilance (mean rank duration) (180 s test)	19.3 ± 3.6^a^ (108.4)	26.5 ± 3.2^a^ (132.6)	45.8 ± 2.2^b^ (162.6)	H = 24.5	<0.001
Vigilance (mean rank duration) (45 s test)	20.7 ± 3.7^a^ (24.8)	26.2 ± 3.4^a^ (29.2)	44.7 ± 2.2^b^ (35.8)	H = 21.2	<0.001
Attention to threat (s) (60 s test)	24.9 ± 2.0^a^	34.7 ± 2.0^b^	36.2 ± 2.0^b^	F = 9.82	<0.001
Attention to threat (s) (45 s test)	19.7 ± 1.5^a^	26.3 ± 1.5^b^	28.0 ± 1.5^b^	F = 8.17	<0.001
Zones crossed (180 s test)	3.1 ± 0.1 (22.6)	3.3 ± 0.1 (26)	3.3 ± 0.1 (26)	*X*^*2*^ = 0.76	0.68
Zones crossed (45 s test)	1.4 ± 0.2^a^ (4.0)	2.2 ± 0.2^b^ (8.0)	2.1 ± 0.1^b^ (8.6)	*X*^*2*^ = 10.9	0.004
Time eating (mean rank duration) (180 s test)	43.4 ± 3.1^a^ (54)	35.6 ± 2.7^a^ (26.7)	12.5 ± 0.0^b^ (0)	H = 36.2	<0.001
Time eating (mean rank duration) (45 s test)	41.3 ± 3.5^a^ (13.9)	34.7 ± 3.2^a^ (7.6)	15.5 ± 0.0^b^ (0)	H = 27.0	<0.001

Different superscripts (^a,b^) within rows indicate a significant difference between treatments as determined using post-hoc analyses. Mean rank durations are given for vigilance and time eating, raw means (s) are given in parentheses. Least-squares means are given on the log scale for zones crossed, back-transformed means are given in parentheses.

**Fig 2:**
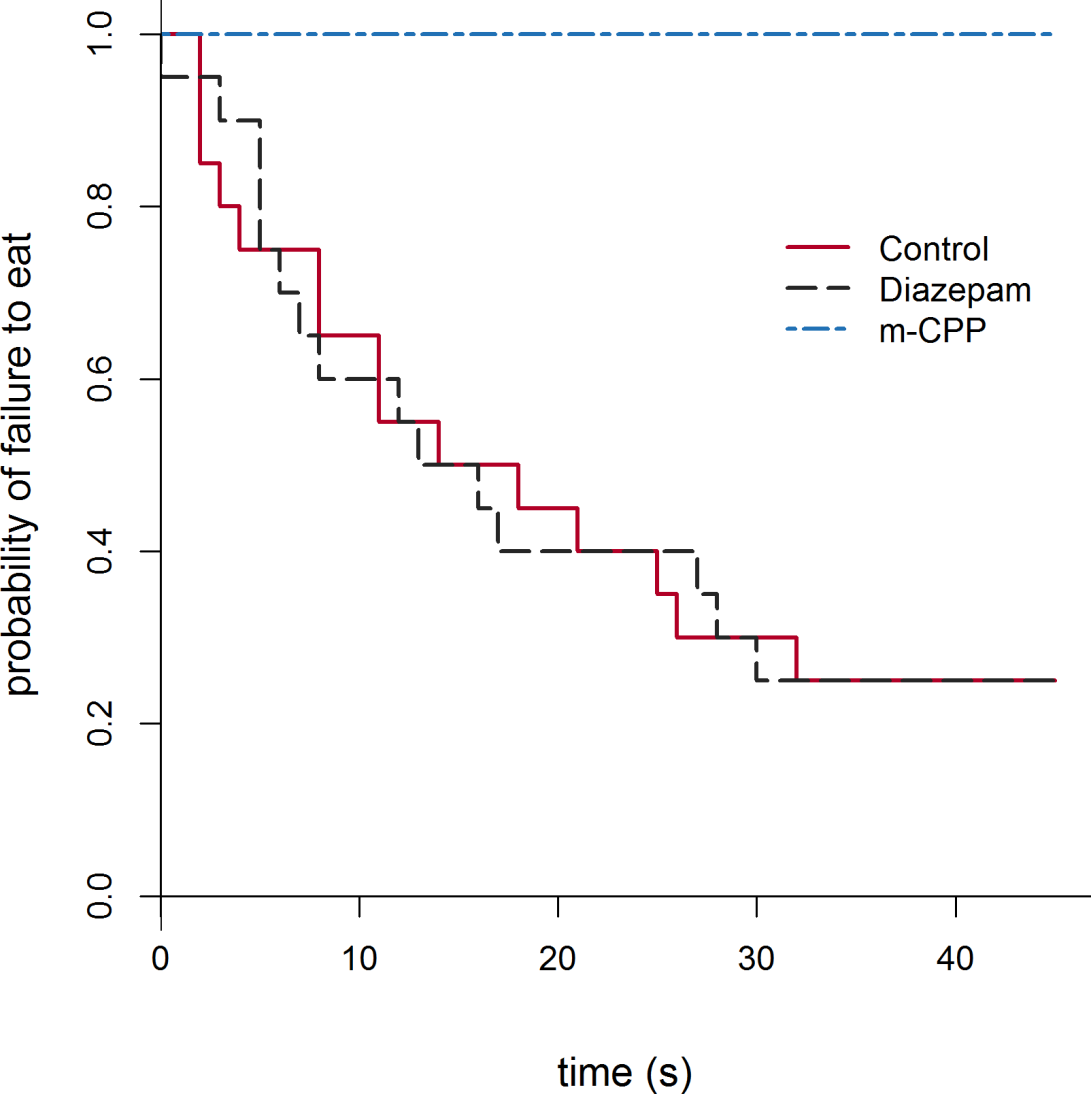
Kaplan-Meier curves for latency to eat during the 45 s time period in experiment 1. Every time an animal initiated its first eating event, the proportion of sheep which failed to eat on the Y axis drops.

No sheep in the m-CPP group fed during the test, while only 2 and 5 animals failed to eat during the 180 s and 45 s time periods respectively for both the Control and Diazepam groups (log-rank P<0.001). The hazard ratios of the Diazepam and Control groups did not differ, indicating they were likely to begin eating the food at a similar rate for both the full length (180 s) and shortened (45 s) tests (Table 2). The Kaplan-Meier plot for the 45 s test shows the time of each animal’s first feeding event and the proportion of sheep which failed to eat (Fig 2). The m-CPP group spent the least time eating while the Diazepam group spent the most time eating, however the difference between the Control and Diazepam groups was not statistically significant (Table 1).

**Table 2:** Hazard ratios for latency to eat in the 180 s and 45 s attention bias test as affected by treatment in experiment 1.

Test duration (s)	Treatment	Coefficient^1^	SE (coeff)	Hazard ratio^2^	P value
**180**	Control	Reference			
	Diazepam	0.04	1.04	1.04 (0.51–1.57)	0.9
**45**	Control	Reference			
	Diazepam	0.00	0.37	0.99 (0.54–1.60)	0.99

Hazard ratios indicate likeliness to eat the feed compared to the reference treatment. A hazard ratio <1 indicates a reduced hazard, >1 indicates an increased hazard, 1 = no effect.

^1^ Regression coefficient from the Cox-proportional hazards model

^2^ 95% CI in parentheses.

### Experiment 2

The No-dog group spent significantly less time displaying vigilance behaviour than the Dog and Logger groups during the 180 s test (Table 3). However, a significant difference was no longer seen between the Dog and No-dog groups when the test was shortened to 60 or 45 s (observed differences 10.12 and 7.1 respectively < critical difference 13.22). Vigilance did not differ between the Dog and Logger groups at any time period. Attention to threat did not differ between any treatment groups (Table 3). Sheep in the No-dog group spent the most time eating. While an overall treatment effect was found for time eating (P = 0.03), the observed differences between the No-Dog vs. the Dog (12.0) and Logger (12.7) groups were less than the critical difference (13.22) in post-hoc analyses (Table 3).

**Table 3:** Mean ± s.e.m. behavioural responses of sheep during the attention bias test in experiment 2.

Behavioural measure	No-dog	Dog	Logger	Test value	P value
Vigilance (mean rank duration) (180 s test)	20.0 ± 3.0^a^ (147.0)	35.7 ± 3.6^b^ (165.4)	35.9 ± 4.1^b^ (163.3)	H = 11.0	0.004
Vigilance (mean rank duration) (60 s test)	22.4 ± 3.8^a^ (51.2)	32.5 ± 3.3^ab^ (56.0)	36.7 ± 4.0^b^ (56.1)	H = 7.2	0.03
Vigilance (mean rank duration) (45 s test)	24.1 ± 4.1 (38.9)	31.2 ± 3.3 (42.4)	36.2 ± 3.9 (42.6)	H = 5.0	0.08
Attention to threat (s) (60 s test)	38.1 ± 1.8	39.5 ± 1.8	40.7 ± 1.8	F = 0.48	0.62
Time eating (mean rank duration) (180 s test)	38.7 ± 3.5 (9.6)	26.7 ± 3.7 (2.7)	26.1 ± 3.7 (3.0)	H = 7.0	0.03

Different superscripts (^a,b^) within rows indicate a significant difference between treatments as determined using post-hoc analyses. Mean rank durations are given for vigilance and time eating, raw means (s) are given in parentheses.

**Fig 3:**
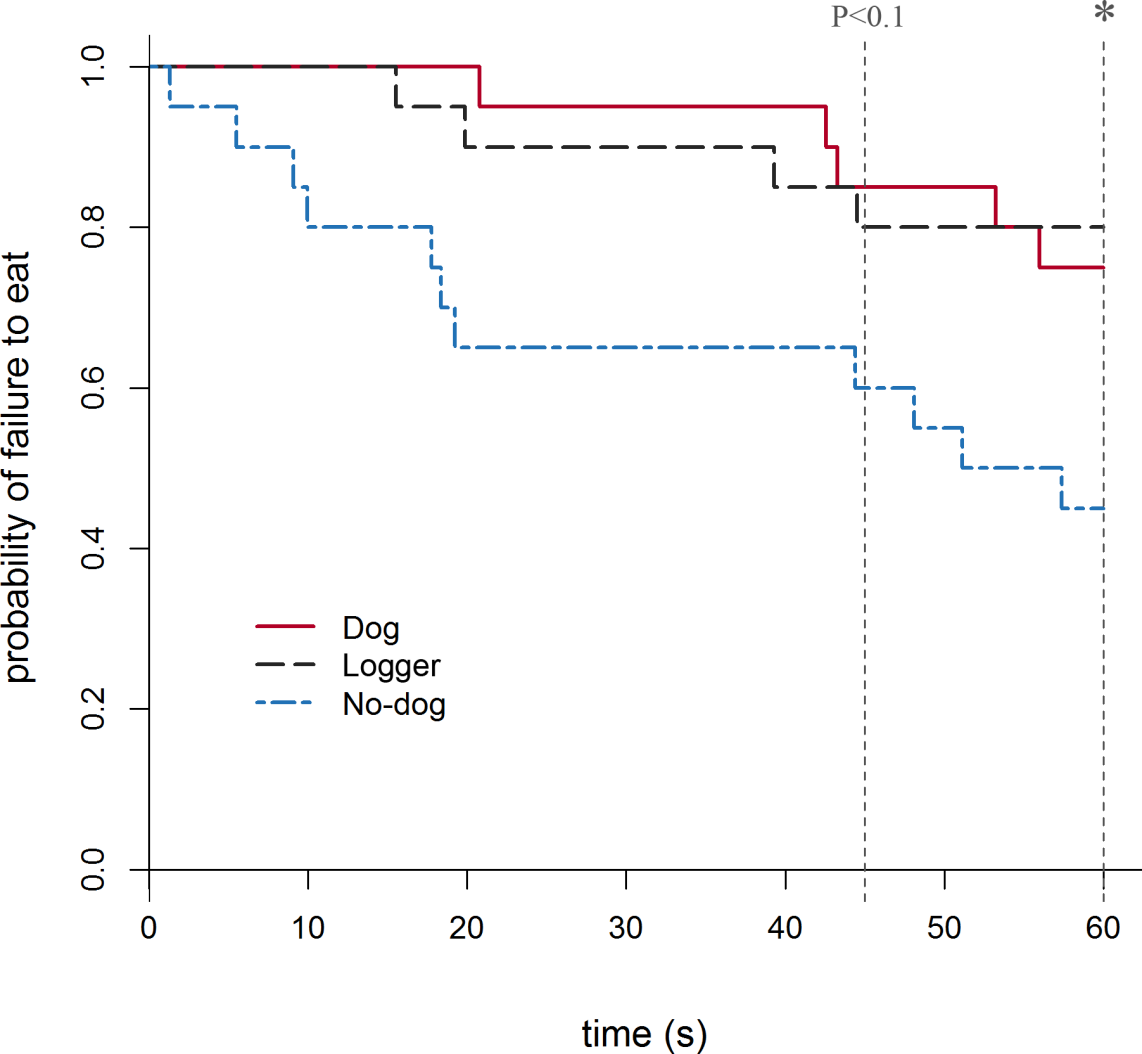
Kaplan-Meier curves for latency to eat during the 60 s time period in experiment 2. Every time an animal initiated its first eating event, the proportion of sheep which failed to eat on the Y axis drops. The effect of treatment group was significant at 60 s (*) and tended towards significance at 45 s (P<0.1).

The hazard ratios for the Dog and Logger groups did not differ, indicating they were likely to first eat the food at a similar rate for both the full length (180 s) and shortened (45 s) tests (Table 4). For the 180 s test, the No-dog group was approximately 3 times more likely to eat the hay than the Dog group (P = 0.007). This was also evident when the test was shortened to 60 s (P = 0.042), however when shortened to 45 s there was only a tendency for the Dog and No-dog hazard ratios to be different (P = 0.066). The Kaplan-Meier plot for the 60 s test shows the time of each animal’s first feeding event and the proportion of sheep that failed to eat (Fig 3).

**Table 4:** Hazard ratios for latency to eat in the 180, 60 and 45 s attention bias tests as affected by treatment in experiment 2.

Test duration	Treatment	Coefficient^1^	SE (coeff)	Hazard ratio^2^	P value
**180**	Dog	Reference			
	No-dog	1.05	0.39	2.87 (1.33–6.19)	0.007
	Logger	0.03	0.43	1.03 (0.45–2.37)	0.95
**60**	Dog	Reference			
	No-dog	1.10	0.54	3.00 (1.04–8.64)	0.042
	Logger	-0.19	0.67	0.83 (0.22–3.08)	0.777
**45**	Dog	Reference			
	No-dog	1.25	0.68	3.48 (0.92–13.14)	0.066
	Logger	0.33	0.76	1.38 (0.31–6.18)	0.671

Hazard ratios indicate likeliness to eat the feed compared to the reference treatment. A hazard ratio <1 indicates a reduced hazard, >1 indicates an increased hazard, 1 = no effect.

^1^ Regression coefficient from the Cox-proportional hazards model

^2^ 95% CI in parenthesis.

When excluding the two outliers, the number of zones crossed were higher for the No-dog group than the Dog group (t = 2.81, P = 0.007), however when outliers were included in the model this was not significant (t = 1.42, P = 0.16). Zones crossed did not differ between the Dog and Logger groups (t = 0.54, P = 0.59). More animals in the No-dog group sniffed the window cover than the Dog and Logger groups (11, 3 and 3 animals respectively, fishers exact test, P = 0.008).

## Discussion

The results of the current study support our initial hypotheses that 1) sheep would be willing to eat a novel feed during the test, 2) the test duration could be shortened from 180 s, 3) the behaviours measured in the test were at least partially a response to the dog and 4) attachment of data loggers prior to testing did not alter animal behaviour in the test. Experiment 1 demonstrated that sheep do not require training to a feed bucket prior to testing, as 36 of 40 animals in the Control and Diazepam groups ate the novel feed presented in the test. This means the test can be conducted in a single day, making it a more practical measure of anxious states in sheep. It should however be noted that the sheep were withheld from feed overnight prior to testing, which may be necessary so they are motivated to eat, and to reduce variability in appetite during the test. Furthermore, the treatment differences for vigilance, latency to eat and attention to threat were consistent across the 45 s and 180 s time periods, indicating the test can potentially be shortened to less than 1 min per sheep. This allows for a greater number of animals to be tested across a day, making the test more applicable to larger populations.

Sheep exposed to a dog were more vigilant and less likely to eat in the attention bias test than those which were exposed to an empty window, supporting our hypothesis that the behaviours measured in the test were at least partially a response to the dog. During the first 60 s of the test however, this effect was not significant which may indicate other stimuli, such as sudden window cover movement, contribute to the responses of sheep during the test. Given the results of experiment 1 and Lee et al. [13], which demonstrate the measures can be used to differentiate anxious states, we expect other stimuli related to the test may have also contributed to an anxious state, but to a lesser degree than the presence of a threat. Attention to threat did not differ between any treatment groups, indicating this measure did not discriminate between a response to the dog and a response to sudden window cover movement. Once again this may indicate sudden movement causes an anxious response in animals which is initially indistinguishable from that caused by the presence of a predator using this test paradigm.

There were no differences between treatment groups for zones crossed and vocalisation counts during the 180 s test, although the Diazepam group crossed fewer zones during the first 45 s of testing. Decreased activity relative to the other treatment groups may indicate Diazepam had a sedative effect on the sheep, however we expect it is more likely that animals in the Diazepam group crossed fewer zones because they chose to spend more time eating, which directly competed with total time available to cross zones during the test. Within the concept of affect being the position of an animal in a two dimensional space described by axes of valence and arousal [1,2], we propose zones crossed in this study may be closer associated with arousal than valence. If this interpretation is correct, arousal does not appear to have been strongly influenced by pharmacologically heightened anxiety or exposure to the dog in the attention bias test. In contrast, heightened anxiety resulted in enhanced vigilance and increased latency to eat which are likely to be associated with a negatively valenced state. This finding may create the potential for the test to assess the valence of an affective state independent of arousal. Alternatively, zones crossed may not have been a suitable measure of arousal in this study, in which case we cannot be sure whether the pharmacological treatments modified arousal. Measurement of physiological responses during the test in future studies may help to better assess the arousal dimension of affective state and help determine the potential for the test to discriminate between arousal and valence.

During experiment 1, many sheep within the m-CPP treated group displayed abnormal behaviours such as head, tail and whole body shaking, indicating an adverse reaction to the drug. The same dose rate did not cause an adverse response in adult ewes [13] but has elicited abnormal behaviours in 8-month-old sheep [16], indicating this dose rate may be inappropriate for younger animals. We propose the undesirable responses in this study had minimal impact on vigilance, or alternatively may have further increased anxiety and therefore vigilance due to the compromised ability of sheep to escape [24]. In each case, vigilance should still be a valid indicator for anxiety in the attention bias test. This is supported by previous studies in starlings, sheep and humans which consider vigilance to be a key measure for attention bias [12,13,25]. Attention to threat did not differ between the Control and m-CPP groups, which could potentially be related to the adverse response to the drugs if m-CPP treated sheep were disoriented. However, this measure should still be valid for the Control and Diazepam groups which differed significantly. This is supported by the findings of Lee et al. [13], showing attention to threat may be a key measure in the attention bias test.

The drugs used in this study have been known to effect feeding behaviour, with m-CPP dose-dependently suppressing food intake in rodents and humans [26–29] and diazepam increasing food intake in birds and non-human primates [30,31]. An adverse response to the drug may have further impacted appetite and feeding behaviours in the m-CPP group. Consequently, latency to eat and total time spent eating cannot be considered reliable measures for experiment 1. This is not to say latency to eat cannot be a useful indicator in the attention bias test in the absence of drug treatments. Latency to eat was a key measure of attention bias for starlings [12] and the results from experiment 2 where no drug treatments were given indicate feeding behaviours are directly related to the presence of the dog. If our interpretation that latency to eat is primarily an assessment of valence rather than arousal is valid, then we can conclude that latency to eat may be a key measure of valence within the attention bias test, however further validation is required to confirm this, where anxious state and arousal are independently manipulated.

The current study presents a quick, easy method of data logger attachment which does not appear to significantly impact animal behaviour in the test. As collation of behavioural data is often time consuming and labour intensive, automation allows for more rapid and practical tests including, but not limited to, the attention bias test. This is of particular importance if the test is to be applied to large groups of animals. While this study focused on automation of vigilance behaviour, data loggers can potentially be used for automation of the other key measures in the test such as attention to threat or latency to eat. Importantly, data loggers may also help further determine the role of arousal in modifying performance of animals in the test.

This study provides further pharmacological validation that the attention bias test may be useful for detecting anxious states in sheep. We have also demonstrated that the test may be useful across different ages and sexes, as the current study tested young castrated males while Lee et al. [13] tested adult ewes. While these results are promising, the attention bias test is still new and more work is required to better understand, validate and refine the test. We suggest a number of priority areas to begin further work. Firstly, while most studies in humans have only found attention biases in anxious individuals, there is evidence that attention biases also occur in clinically depressed individuals [32,33]. Further studies could assess whether other negative affective states, such as depression, can result in attention biases in animal species detectable with this test paradigm. Such studies should also help clarify the extent to which the test is primarily a measure of valence. Second, further refinement of the method is required for the test to become practical for large groups of animals or for use in an on-farm setting. Automation of behavioural measures and adapting the test to work in existing sheep handling facilities are two routes which would make this test more practical. Finally, there is potential for the attention bias test to be used as a measure of temperament as well as a measure of transient anxious states, however further research is required to explore this potential. If the test can be used to assess an anxious trait, it could be applied to larger groups of animals for estimation of genetic parameters related to anxious behaviours and temperament. Anxious temperament is known to be influenced by genetic factors and temperament is a heritable trait [34–36]. By identifying and incorporating temperament into sheep breeding programs, we may be able to select for calmer animals that are better suited to a domestic environment, are easier to handle and have improved welfare.

## Conclusions

Overall, the current study shows the attention bias test developed by Lee et al. [13] can be further refined so that it does not require training and may be shortened to less than 1 min per animal. This faster method for assessing anxious states in sheep may provide a more practical measure of affect which can be used in further animal welfare research. This study also verifies the responses being measured in the test are at least partially a response to the dog, further validating it as a measure of attention bias. The potential for the test to discriminate valence from arousal deserves closer examination. With further refinement and automation the test should be suitable for application to larger populations of animals.

## Supporting information

S1 Video Video showing the beginning of the test and examples of key behaviours. (MP4)

S1 Dataset Raw behavioural data for experiment 1. (XLSX)

S2 Dataset Raw behavioural data for experiment 2. (XLSX)
